# Beyond Mean HU: Spatial Radiodensity Mapping of the Human Hyoid as a Baseline for Morphological and Biomechanical Investigation

**DOI:** 10.3390/biology15181602

**Published:** 2026-09-11

**Authors:** Alexander Morávek, Katarína Martinková, Petr Henyš, Jana Velemínská, Niels Hammer, Michal Kuchař

**Affiliations:** 1 Department of Anatomy, Faculty of Medicine in Hradec Kralove, Charles University, 50003 Hradec Kralove, Czech Republic; 2 Department of Histology and Embryology, 3rd Faculty of Medicine, Charles University, 10000 Prague, Czech Republic; 3 Department of New Technologies and Applied Informatics, Faculty of Mechatronics, Informatics and Interdisciplinary Studies, Technical University of Liberec, 46117 Liberec, Czech Republic; 4 Department of Anthropology and Human Genetics, Faculty of Science, Charles University, 12800 Prague, Czech Republic; 5 Division of Macroscopic and Clinical Anatomy, Gottfried Schatz Research Center, Medical University Graz, 8036 Graz, Austria; 6 Department of Orthopaedic and Trauma Surgery, University of Leipzig, 04109 Leipzig, Germany; 7 Division of Biomechatronics, Fraunhofer Institute for Machine Tools and Forming Technology (IWU), 09126 Chemnitz, Germany

**Keywords:** bone heterogeneity, computed tomography, hyoid bone, image registration, radiodensity mapping, spatial phenotyping, supervoxel analysis, template-based mapping, age-related variation, sexual dimorphism

## Abstract

Bone radiodensity is often reduced to global Hounsfield Unit (HU) summaries, although its spatial distribution may contain biologically relevant information. This study introduced a template-based framework for spatial radiodensity mapping of the adult human hyoid and tested whether spatial descriptors capture age- and sex-related variation better than whole-bone HU measures. Clinical head-and-neck CT scans from 300 adults, including 180 females and 120 males, were analyzed. Hyoids were segmented, normalized into a common anatomical template, and divided into 1500 supervoxels for spatial analysis. Scalar HU summaries were compared with PCA-based spatial descriptors, and regional effects were assessed using supervoxel-wise models with false-discovery-rate correction. The registered radiodensity maps showed spatial coherence, with neighboring supervoxels displaying more similar profiles than distant ones. In this cohort, spatial descriptors outperformed mean HU for age prediction, although performance remained limited (R^2^ = 0.255). Sex classification was moderate (ROC AUC = 0.810; balanced accuracy = 0.725). After the spatial features were adjusted for native volume, sex-classification AUC fell to 0.671, and none of the 51 unadjusted regional sex associations survived volume adjustment. Age-related associations involved the hyoid body, the body–greater horn transition, and the proximal-to-mid greater horns. These findings demonstrate spatial coherence in the registered maps but show that the sex-related pattern is strongly size-dependent.

## Simple Summary

A single average CT value can conceal regional differences within a bone. We mapped clinical CT radiodensity across anatomically corresponding regions of 300 adult hyoids (19–84 years) after aligning each bone to a common template. Nearby regions had more similar radiodensity profiles than distant regions, and in this cohort, spatial descriptors retained more age-related information than whole-bone summaries. Absolute age-prediction performance remained limited; native hyoid volume explained much of the sex-related signal, although volume-adjusted spatial discrimination remained above chance. Age-related regional associations involved the hyoid body, the body–greater horn transition, and parts of the greater horns. Because the study is cross-sectional and uses uncalibrated CT, these maps neither measure mechanical loading nor establish a clinical or forensic test. They provide an exploratory anatomical baseline and candidate regions for future fusion-aware, calibrated, and biomechanical validation.

## 1. Introduction

Bone is a dynamic and spatially heterogeneous tissue whose structure is continuously shaped by development, ageing, systemic physiology, and local mechanical demands. According to the mechanostat concept and broader principles of bone functional adaptation, skeletal tissue responds to its mechanical environment through regionally regulated modeling and remodeling processes [1,2,3]. Consequently, bone mineralization, cortical thickness, trabecular organization, and tissue-level properties are not expected to be uniform within a skeletal element. However, many imaging-based studies still reduce this heterogeneity to global values such as mean radiodensity, total volume, or predefined regional averages [4,5,6]. Although such summaries are useful, they may obscure biologically relevant regional variation that is important for structure–function interpretation. Although this study did not directly examine applied mechanical loading, it is based on established concepts regarding the effects of forces and surrounding tissues on the hyoid bone itself.

Clinical computed tomography (CT) offers an opportunity to examine skeletal radiodensity in vivo and in retrospective datasets. Routine CT scans contain voxel-wise attenuation values expressed in Hounsfield Units, which can be used to describe relative radiodensity throughout an entire bone [7,8,9]. These values are not equivalent to calibrated bone mineral density and are influenced by scanner model, acquisition parameters, tube voltage, reconstruction kernel, spatial resolution, and beam-hardening effects [6,9,10,11]. Nevertheless, when interpreted cautiously as relative radiodensity within a controlled analytical framework, clinical CT can provide spatial information about internal skeletal organization.

A central challenge in spatial bone phenotyping is anatomical correspondence. Bones differ in size, orientation, curvature, fusion status, and local morphology, making it difficult to compare apparently similar locations across individuals [12,13,14,15]. This problem is particularly important for small and morphologically variable skeletal elements, where manually defined regions of interest may be inconsistent and global measures may average across functionally distinct regions. Template-based anatomical normalization addresses this limitation by mapping individual bones into a common coordinate system, enabling region-by-region comparison of radiodensity across subjects.

The human hyoid bone appears well suited to this approach. It has no direct osseous articulation and is suspended within a complex muscular and ligamentous system connecting the mandible, skull base, tongue, larynx, sternum, and scapular region. It participates in swallowing, speech, hyolaryngeal elevation, and upper airway function, and its movement depends on coordinated activity of multiple supra- and infrahyoid muscles [16,17,18,19,20]. Dynamic CT has shown that different hyoid-related muscles contribute differently to upward and forward components of hyoid movement during swallowing, indicating that force transmission through the hyoid is spatially and temporally organized rather than uniform [19]. The hyoid therefore represents a small but functionally informative skeletal element in which regional structural variation may reflect the combined effects of ageing, sex, fusion status, and local functional demands. Although the hyoid is not a weight-bearing bone in the classical sense, it is mechanically important as a mobile force-transmission element within the supra- and infrahyoid muscular and ligamentous system. During swallowing and related upper aerodigestive functions, different muscle groups contribute to superior, anterior, and laryngeal-coupling components of hyoid movement rather than loading the bone uniformly. Such directionally and temporally heterogeneous loading provides a functional rationale for expecting local, rather than whole-bone uniform, variation in hyoid internal structure. If CT-derived radiodensity partly reflects local mineralized tissue organization, then preserving its anatomical distribution may reveal structure–function information that is not accessible from a single mean HU value. This is particularly relevant for the hyoid, where muscle and ligament attachments, fusion of the body with the greater horns, and sex- and age-related skeletal changes may interact within a small and morphologically variable bone.

Previous anatomical, forensic, and CT-based studies have shown that hyoid morphology, fusion, and ossification vary with age and sex [18,20,21,22,23,24,25]. CT-derived hyoid radiodensity has also been reported to be lower in adult females than males and to decrease with age in adulthood [26,27]. Micro-CT studies further show that the hyoid body and greater horns differ in internal structure, including bone volume, bone density, bone volume fraction, trabecular connectivity, and trabecular distribution, with ossification changes at the body–greater horn junction [28]. In addition, bone-quality analyses indicate that regions related to infrahyoid muscle and ligament entheses may differ in biological apatite crystallite orientation even when regional BMD differences are not significant [29]. These findings suggest that hyoid variation is not only global or morphometric, but also regional and tissue-level. However, most population-level CT studies of the hyoid have relied on whole-bone or single-region radiodensity measurements, fusion or ossification scores, and morphometric descriptors rather than spatially resolved radiodensity maps [20,26,27,30,31]. Although spatial QCT and cortical bone mapping studies in other skeletal elements have shown that regional information can be lost in global density measures, it remains unclear whether CT-derived hyoid radiodensity shows a spatial whole-bone pattern, and whether this pattern captures age- and sex-related information that mean HU values miss [15,32,33].

In this study, we introduce a template-based framework for spatial radiodensity mapping of the adult human hyoid from routine clinical CT. We hypothesized that, after anatomical normalization, hyoid radiodensity would exhibit spatial organization consistent with a regionally structured radiodensity phenotype and that spatial descriptors would capture age- and sex-related variation more effectively than conventional whole-bone HU summaries. Specifically, we aimed to construct a common hyoid template, map voxel-wise CT radiodensity into template space, evaluate distance-dependent spatial structure, compare scalar and spatial feature representations, and localize regional age- and sex-associated radiodensity patterns. The study is therefore designed as an associative anatomical and statistical baseline. Localized regions are treated as candidates for future calibrated, fusion-aware, morphological, and biomechanical validation rather than as evidence of mechanically caused remodeling.

## 2. Materials and Methods

### 2.1. Study Design, Cohort, and Hyoid Segmentation

The present study was approved by the Institutional Review Board of Charles University, Faculty of Science (approval no. 2024/17, approved on 10 August 2024). All individuals provided signed informed consent for participation in long-term scientific research.

The study was based on fully anonymized clinical head-and-neck computed tomography (CT) examinations of adults. All CT examinations were acquired in routine clinical practice using a Siemens SOMATOM Definition Flash scanner (Siemens Healthcare, Forchheim, Germany) operated at 120 kV, with reconstruction using the H60s kernel and a voxel size of 0.508 mm × 0.508 mm × 0.600 mm.

Only scans that allowed reliable hyoid segmentation and subsequent spatial analysis were included. Examinations were excluded in the presence of major motion or reconstruction artifacts, fractures in the hyoid region, grossly visible pathological changes on initial visual inspection, incomplete visualization of the hyoid, or any other finding likely to compromise anatomical segmentation or radiodensity analysis. The final study sample comprised 300 adults aged 19–84 years, including 180 females and 120 males. The overall mean age was 45.57 ± 13.80 years, with a mean age of 46.74 ± 13.41 years in females and 43.81 ± 14.24 years in males.

Because the study was based on uncalibrated clinical CT scans acquired on a single CT scanner without a calibration phantom and assembled from temporally separated data collections, acquisition-related technical variation was accounted for in the statistical analyses. The final study sample comprised four acquisition phases defined by data source: Phase A (*n* = 128), Phase B (*n* = 73), Phase C (*n* = 70), and Phase D (*n* = 29). The phase-specific age and sex composition was: Phase A, 39.30 ± 8.50 years, 74 females/54 males; Phase B, 40.21 ± 8.96 years, 39 females/34 males; Phase C, 58.76 ± 15.72 years, 46 females/24 males; and Phase D, 54.86 ± 10.61 years, 21 females/8 males.

All hyoid bones were segmented automatically using TotalSegmentator (version 2.12.0) [34]. Segmentation quality was assessed from automatically generated volume-rendered previews in four standard views and subsequently checked manually by the first author (AM), who has previous experience with CT-based hyoid segmentation and template construction. Cases with incomplete visualization of the hyoid, major motion or reconstruction artifacts, or visibly incorrect or incomplete segmentation were excluded. Of 368 examined cases, 300 were retained for further analysis. Segmentation accuracy was additionally evaluated in all 74 source-verified cases for which manual reference masks from a previous project were available. Agreement between automated and manual segmentations yielded a Dice Similarity Coefficient (DSC) of 0.751 ± 0.150 and an HD95 of 3.37 ± 6.61 mm. The TotalSegmentator masks included the hyoid body, greater horns, lesser horns, and the body–greater horn junction as one continuous anatomical structure. Original CT values were retained throughout this entire region regardless of tissue composition, whereas voxels outside the hyoid mask were replaced by artificial background. Fusion at the body–greater horn junction was not staged separately. Accordingly, the registration procedure treated the entire junction as part of the hyoid in all cases, irrespective of whether osseous union was absent, partial, or complete.

### 2.2. Template Construction and Spatial Mapping

A population-based hyoid template was constructed from the segmented volumes using a multistage registration pipeline consisting of rigid, affine, and bijective diffeomorphic transformations performed with the ANTsPy (version 0.6.1) SyNRA preset [12], following a previously published pipeline [35,36] and methodological workflow [37]. The template was constructed once from all 300 included segmentations before cross-validation and was not rebuilt within individual folds; its construction used anatomical geometry without reference to age, sex, or predictive outcomes. Individual hyoid bones were then warped into a common anatomical space while preserving smooth one-to-one correspondence between each subject and the template. The non-linear registration stage used a Demons metric with (1200, 1200, 1200, 1200) iterations. Full registration parameters are provided in the accompanying configuration file available on Figshare. After shape normalization, voxel-wise Hounsfield Unit (HU) values were transferred from each subject’s CT image into template space onto spatially corresponding registered points using linear interpolation. A template mask was then used to define the anatomically applicable hyoid region for subsequent analyses. This common template space also enabled direct visualization of spatial statistical outputs on the hyoid surface or volume, thereby facilitating anatomical interpretation of regional patterns; such maps can be readily explored in standard 3D visualization software such as ParaView (version 6.0.1) [38].

### 2.3. Whole-Bone and Supervoxel Feature Extraction

Within the hyoid bone, voxels were considered valid when the HU value was finite and ≥−850. This threshold was chosen as a practical compromise between removing background noise and preserving most of the relevant anatomical information. This threshold was not intended to define mineralized bone tissue; the anatomical region was already defined by the hyoid mask. It was used only to remove background, padding, and extreme resampling artifacts introduced during warping. Because the study relied on uncalibrated clinical CT data, HU values were interpreted primarily as a within-cohort comparative signal reflecting relative internal radiodensity structure rather than as absolute densitometric measurements.

For each individual, whole-bone summary measures were calculated across all valid voxels, including mean HU, median HU, standard deviation, lower and upper distribution percentiles (10th, 25th, 50th, 75th, and 90th percentiles), and the number of valid voxels. To retain spatial structure, the template-space hyoid was further partitioned into 1500 supervoxels using MiniBatchKMeans clustering of voxel coordinates. This coordinate-defined partition was likewise generated once in the full-sample template space and was not rebuilt within individual cross-validation folds. The k = 1500 partition was selected before inspection of age- or sex-related results and was retained as the primary reference setting rather than as a data-driven optimum. Robustness was subsequently assessed by repeating the regional analyses with k = 1000 and k = 2000 and comparing the resulting coefficient maps after expansion to template voxels. HU values within each supervoxel were aggregated by the arithmetic mean, yielding a subject-by-supervoxel matrix. Missing values were retained during feature extraction and were imputed by column means only in analyses that required a complete matrix. As a multivariate sensitivity analysis, the raw-PCA prediction models were repeated using five-nearest-neighbor imputation. Both imputation methods were fitted exclusively within each training fold and then applied to the corresponding held-out data. Regional models used the available observations at each supervoxel without imputation.

### 2.4. Dimension Reduction and Batch Harmonization

The supervoxel matrix was reduced by principal component analysis (PCA). The number of retained components was determined by cumulative explained variance, with a target of 90% and a maximum of 40 principal components. The maximum of 40 components was specified a priori as a computational cap and was not treated as an optimized value. In the present dataset, the retained 40 components explained 85.27% of the total variance. Batch harmonization was then performed using ComBat to reduce acquisition-related HU shifts while preserving age- and sex-related biological variation; age and sex were included as biological covariates. The technical batch variable combined acquisition phase with reconstruction-selection status; when multiple kernel reconstructions were available for the same examination, the single reconstruction matching the H60s kernel used in this study was selected for analysis. Harmonization was applied both to the whole-bone summary features and to the PCA-derived feature space and was performed in mean-only mode. Importantly, the regional supervoxel-wise models were fit to the original supervoxel mean HU matrix rather than to ComBat-harmonized supervoxel values. In these regional models, age, sex, and technical variables were included directly as covariates. Raw and ComBat-adjusted feature representations were evaluated separately in the cross-validated predictive analyses. Regional localization was based on raw supervoxel HU values, with technical variables included directly as model covariates, thereby retaining the original HU scale for local effect estimates. These regional coefficients were not interpreted as contributions to PCA-based predictive performance. Their robustness was additionally assessed by repeating the regional models after mean-only ComBat harmonization, with age and sex preserved as biological covariates. Within each cross-validation fold, ComBat was likewise fitted exclusively on the training data, with the combined acquisition phase and reconstruction-selection status as the batch variable and age and sex included in the training design matrix. Held-out data were transformed using the stored training estimates and the corresponding technical batch label only; held-out age and sex were neither supplied nor used.

### 2.5. Statistical Analysis and Predictive Modeling

To test whether hyoid radiodensity is better represented by global descriptors or by internal spatial organization, we compared five feature sets: mean HU alone, raw whole-bone summary features, ComBat-adjusted summary features, raw PCA scores derived from supervoxels, and ComBat-adjusted PCA scores. Age-related variation was modeled using ridge regression, whereas sex-related discrimination was evaluated using class-weighted logistic regression. Predictors were standardized before model fitting, and performance was estimated using five-fold cross-validation, with standard K-fold splitting for age and stratified K-fold splitting for sex. Missing-value imputation and PCA were fitted within the training folds and then applied to the corresponding held-out folds. ComBat was likewise fitted exclusively on the training data, with the combined acquisition phase and reconstruction-selection status as the batch variable and age and sex included in the training design matrix. Held-out data were transformed using the stored training estimates and the corresponding technical batch label only; held-out age and sex were neither supplied nor used. Age models were evaluated using mean absolute error, root-mean-square error, R^2^, and Spearman correlation; sex models were evaluated using ROC AUC, balanced accuracy, sensitivity, and specificity. Across-fold variability was summarized by the standard deviation of each metric over the five held-out folds. Mean-HU and raw-PCA performance was compared using exact paired Wilcoxon tests across folds; subject-level out-of-fold age errors were also compared using a two-sided paired Wilcoxon test, and the AUC difference was assessed using a paired stratified bootstrap with 5000 resamples.

Spatial structure within the supervoxel representation was assessed by calculating Pearson and Spearman correlations between supervoxel radiodensity profiles across subjects and relating these correlations to Euclidean distances between supervoxel centroids in template space. Correlations were summarized across distance bins using running means and standard deviations. Whole-bone summary measures were analyzed using ordinary least squares models including age, sex, and technical covariates. To formally compare age-related slopes between sexes, an additional supervoxel-wise model included an age × sex interaction together with the same technical covariates; interaction *p* values were subjected to the same BH-FDR procedure. The influence of subgroup size was assessed by repeating the female regional model in 500 phase-stratified random subsamples of *n* = 120, with BH-FDR correction applied within each replicate. Supervoxels with data from fewer than 10 subjects were excluded. Regression coefficients and two-sided *p* values were obtained for each model, and multiple testing was controlled using the Benjamini–Hochberg false discovery rate procedure, with q < 0.05 considered statistically significant. PCA-derived feature sets were used only for cross-validated comparisons and were not interpreted component-wise. Native hyoid volume was calculated from each binary native-space mask as foreground voxel count multiplied by voxel volume, and surface area was obtained from its 0.5 isosurface; Pearson and Spearman correlations of PC1–PC5 with volume and surface-to-volume ratio were calculated, and sex analyses were repeated using volume alone, PCA plus volume, PCA features residualized for volume within each training fold, and regional sex models including volume as an additional covariate.

All analyses were performed in Python 3.13, using statsmodels 0.14.6 for ordinary least squares models, NeuroCombat 0.2.12 for batch harmonization, and scikit-learn 1.8.0 for dimensionality reduction, clustering, predictive modeling, and cross-validation. For supervoxel-wise sex coefficients, sex was coded as female = 0 and male = 1. Therefore, the sex coefficient represents the male–female difference.

## 3. Results

### 3.1. Whole-Bone Radiodensity Characteristics

Across the whole bone, mean radiodensity was 330.85 ± 121.19 HU (range, −42.08 to 611.99 HU; IQR, 247.06–423.21 HU), while median radiodensity was 324.08 ± 96.81 HU (range, 85.64 to 701.82 HU; IQR, 252.59–381.65 HU), indicating substantial inter-individual variability despite a relatively stable number of valid voxels per subject. The number of valid voxels per subject was 21,897 ± 274 (range, 20,457 to 22,172; IQR, 21,777–22,086).

**Figure 1:**
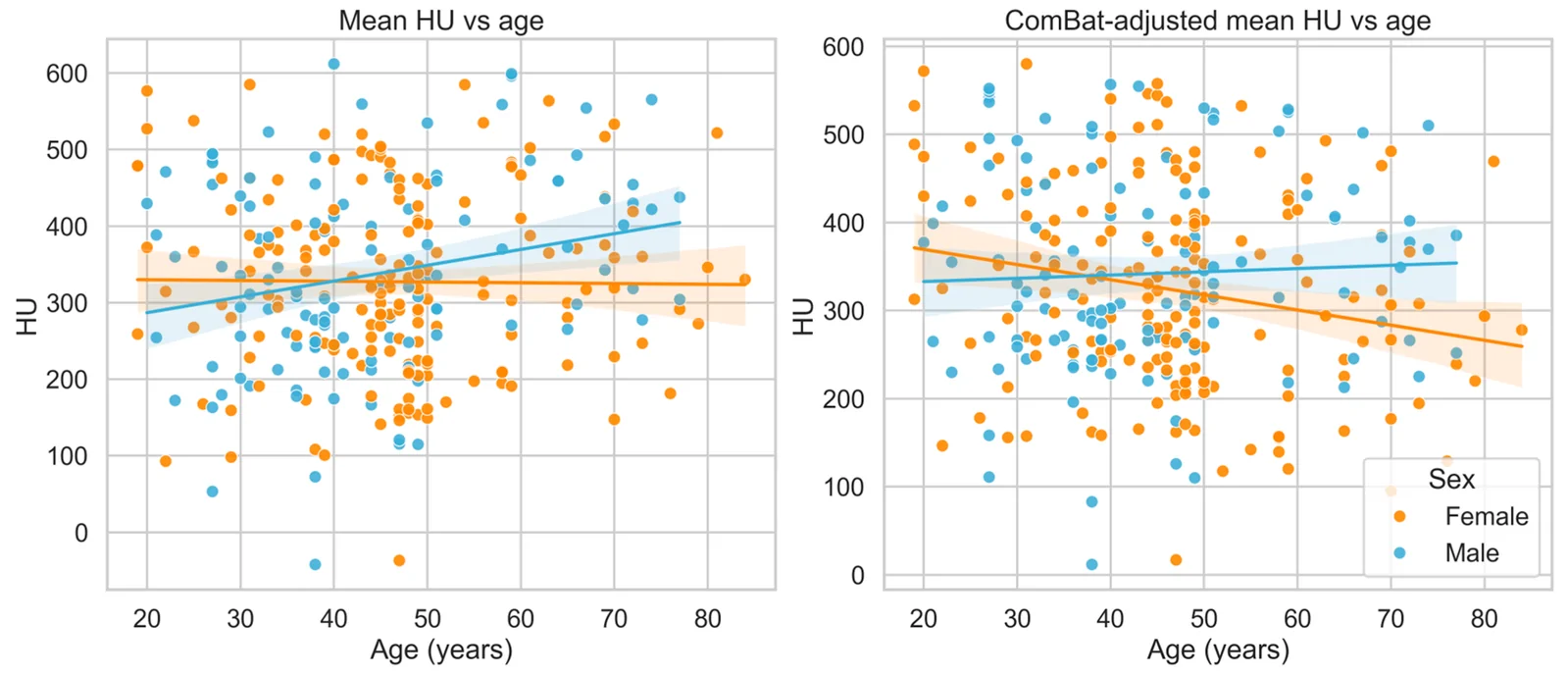
Age-related variation in whole-bone mean hyoid radiodensity before and after ComBat harmonization. Each point represents one individual. Raw CT-derived mean HU values are compared with harmonized values adjusted for acquisition-related technical variation, illustrating the relationship between age and global hyoid radiodensity. Blue and orange lines represent sex-specific regression fits for males and females, respectively; shaded areas indicate 95% confidence intervals.

At the global level, age was associated with lower hyoid radiodensity, whereas sex effects were weak. In the main-effect models, mean HU decreased by 1.22 HU/year (*p* = 0.0467), median HU by 1.15 HU/year (*p* = 0.0192), the 75th percentile by 2.27 HU/year (*p* = 0.0206), and HU standard deviation by 0.80 HU/year (*p* = 0.0409). No convincing sex effect was detected for these whole-bone summary measures. Technical covariates remained relevant at this level, with lower mean and median HU in several parts of the cohort, supporting the need for adjustment and harmonization in subsequent analyses. The relationship between age and whole-bone mean radiodensity, before and after ComBat harmonization, is illustrated in Figure 1.

### 3.2. Supervoxel Representation and Spatial Correlation Structure

Partitioning of the template-space hyoid into 1500 supervoxels yielded a fine-grained spatial representation of the bone, with a mean of 14.83 voxels per supervoxel (median 15; range 4–30). Missingness was limited (579/450,000 values, 0.129%): 1323 supervoxels had *n* = 300, 100 had *n* = 299, 61 had *n* = 298, and 16 had *n* ≤ 297 (median, 300; range, 219–300). Regional models used available cases without imputation (*n* = 219–300 overall, 108–180 in females, and 92–120 in males), well above the prespecified minimum of 10.

**Figure 2:**
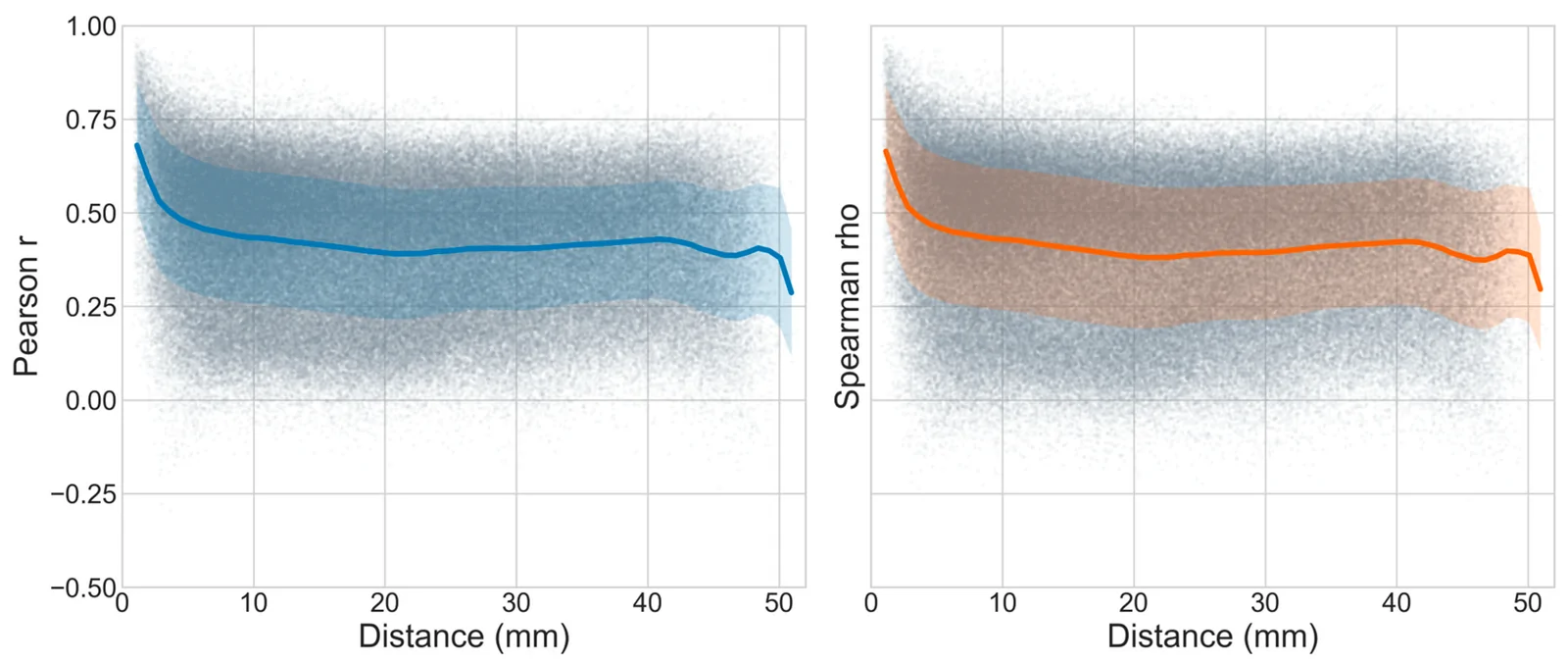
Pairwise supervoxel correlation as a function of Euclidean distance between supervoxel centroids. Each point represents one pair of supervoxels from the full set of pairwise comparisons, with Pearson r shown on the left and Spearman rho shown on the right. The solid line denotes the running mean across distance bins, and the shaded band indicates mean ± 1 SD. Correlations were strongest among nearby supervoxels and declined with increasing distance, indicating local spatial redundancy within the supervoxel representation. Blue and orange lines represent running means for Pearson’s r and Spearman’s ρ, respectively; shaded bands indicate mean ± 1 SD.

Pairwise correlations between supervoxel radiodensity profiles showed a clear distance-dependent structure (Figure 2). Both Pearson and Spearman correlations were strongest among nearby supervoxels and declined with increasing Euclidean distance between supervoxel centroids.

Principal component analysis retained 40 components, explaining 85.27% of the total variance; PC1 alone accounted for 41.80%. After ComBat harmonization, acquisition-related structure was attenuated but not fully removed. Correlations of PC1–PC5 with native volume were 0.108, −0.170, 0.186, −0.268, and −0.129, and those with surface-to-volume ratio were −0.046, 0.251, −0.249, 0.325, and 0.082. After BH correction, PC2–PC5 were associated with volume and PC2–PC4 with surface-to-volume ratio, whereas PC1 was associated with neither measure. Predictive performance generally improved with additional components: for 1/5/10/20/30/40 components, age R^2^ was −0.014/0.030/0.109/0.202/0.250/0.255 and sex AUC was 0.482/0.564/0.584/0.744/0.778/0.810.

### 3.3. Age Prediction from Whole-Bone and Spatial Radiodensity Descriptors

Age-related variation was evaluated across five feature sets: mean HU alone, raw whole-bone summary features, ComBat-adjusted whole-bone summary features, raw PCA features derived from the supervoxel representation, and ComBat-adjusted PCA features.

Although mean HU decreased weakly with age across the cohort (−1.22 HU/year, *p* = 0.0467), mean HU alone did not predict age in held-out individuals (R^2^ = −0.011; Spearman rho = 0.025). The negative cross-validated R^2^ indicates performance slightly worse than predicting the training-fold mean age. Raw whole-bone summary features performed only slightly better (R^2^ = 0.013; Spearman rho = 0.159), whereas ComBat-adjusted whole-bone summary features reached R^2^ = 0.090 and Spearman rho = 0.283.

The PCA-based spatial feature sets showed higher performance than the whole-bone feature sets. The raw PCA representation reached a mean absolute error of 8.91 years, RMSE of 11.89 years, R^2^ = 0.255, and Spearman rho = 0.506. The ComBat-adjusted PCA representation showed similar values, with a mean absolute error of 8.96 years, RMSE of 11.92 years, R^2^ = 0.252, and Spearman rho = 0.506. Raw PCA produced a lower age-prediction error than mean HU in all five folds. The exact paired Wilcoxon test gave W = 0 and *p* = 0.0625, the smallest possible exact two-sided *p*-value with five pairs. At the subject level, raw PCA reduced absolute out-of-fold error by a mean of 1.85 years (95% bootstrap CI, 1.07–2.62 years; two-sided paired Wilcoxon *p* = 9.80 × 10^−6^).

The full comparison of age-model performance across feature sets is presented in Table 1.

### 3.4. Sex Classification from Whole-Bone and Spatial Radiodensity Descriptors

The same comparison was performed for sex-related variation. Mean HU alone showed little to no discriminatory performance between sexes (ROC AUC = 0.471, balanced accuracy 0.481). Whole-bone summary features improved this only moderately (ROC AUC = 0.607, balanced accuracy 0.579), whereas the strongest separation again emerged from the spatial representations. The raw PCA representation reached ROC AUC = 0.810 and balanced accuracy 0.725, while the harmonized PCA representation yielded ROC AUC = 0.808 and balanced accuracy 0.729. The paired stratified bootstrap comparison of mean HU and raw PCA gave an AUC difference of 0.340 (95% CI, 0.259–0.419; *p* < 0.0004). Native volume alone yielded an AUC of 0.909, and adding volume to the raw PCA features yielded an AUC of 0.928. Residualizing the spatial features for volume within each training fold reduced the PCA AUC from 0.810 to 0.671 (difference −0.140; 95% bootstrap CI, −0.198 to −0.082), although performance remained above chance. The full comparison of sex-model performance across feature sets is presented in Table 2.

Replacing training-fold mean imputation with five-nearest-neighbor imputation produced nearly identical predictive performance. For age, mean and KNN imputation yielded R^2^ values of 0.255 and 0.253 and Spearman correlations of 0.506 and 0.502, respectively; corresponding sex-classification results were ROC AUC 0.810 versus 0.815 and balanced accuracy 0.725 versus 0.733. The PCA-based performance advantage was therefore not materially dependent on the selected imputation method. Of the observations retained at ≥−850 HU, 21.35% were below 0 HU and 35.66% were below 150 HU; applying these stricter thresholds yielded age-prediction R^2^ values of 0.233 and 0.231 and sex-classification AUCs of 0.801 and 0.791, respectively. Excluding the six subjects with mean HU below 100 yielded an age R^2^ of 0.221 and a sex-classification AUC of 0.821. In a sensitivity analysis restricted to Phases A and B (*n* = 201), raw PCA retained higher age-prediction performance than mean HU (R^2^ = 0.143 versus −0.007) and higher sex-classification performance (AUC = 0.790 versus 0.402).

### 3.5. Supervoxel-Wise Age and Sex Effects

Regional modeling further showed that age- and sex-related associations were spatially heterogeneous rather than uniformly distributed across the hyoid. In the overall model, 166 of 1500 supervoxels (11.1%) showed a significant age effect after false-discovery-rate correction, with a median age coefficient of −1.07 HU/year. Sex effects were more spatially restricted, with 51 significant supervoxels (3.4%) and a median male–female coefficient of 17.91 HU. Sex-stratified age analyses yielded 314 FDR-significant tests in females and none in males, with median age coefficients of −1.77 and −0.46 HU/year, respectively. However, only 2 of 1500 formal age × sex interaction tests survived FDR correction, with a median interaction coefficient of 1.72 HU/year. In 500 phase-stratified female subsamples of *n* = 120, the median number of significant tests fell to 37 (IQR 0–188), and 27.4% of replicates produced no discoveries, although coefficient directions remained similar to the full female map (median Spearman rho = 0.934). Adding native volume to the regional model reduced the sex findings from 51 to two FDR-significant supervoxels, neither of which belonged to the original set; thus, none of the original 51 associations survived volume adjustment. Native volume itself was significant in 57 supervoxels, and adjustment using log-transformed volume likewise left none of the original 51 sex associations significant. The full comparison of supervoxel-wise regional effects is presented in Table 3.

Across k = 1000/1500/2000, the proportions of FDR-significant tests were 15.0/11.1/12.7% for overall age, 25.7/20.9/19.5% for female age, 0/0/0% for male age, and 4.7/3.4/3.6% for sex. Voxel-expanded coefficient maps showed Spearman correlations of approximately 0.72–0.77 with the primary k = 1500 maps. The broad regional patterns were therefore moderately consistent across granularities, although exact supervoxel boundaries and FDR-significant sets varied with k.

Regional ComBat harmonization produced coefficient patterns broadly similar to those obtained from the primary raw-HU models. Raw-versus-harmonized coefficient-map Spearman correlations were 0.844 for overall age, 0.880 for female age, 0.679 for male age, and 0.996 for sex. Of the FDR-significant findings in the primary models, 126/166 overall-age, 269/314 female-age, and 50/51 sex-associated supervoxels also remained significant after harmonization, while the male-age map changed from no significant findings to one. The overall-age, female-age, and sex patterns were therefore comparatively stable, although the male-age map and exact FDR-significant sets showed greater dependence on preprocessing.

### 3.6. Anatomical Distribution of Regional Age and Sex Effects

**Figure 3:**
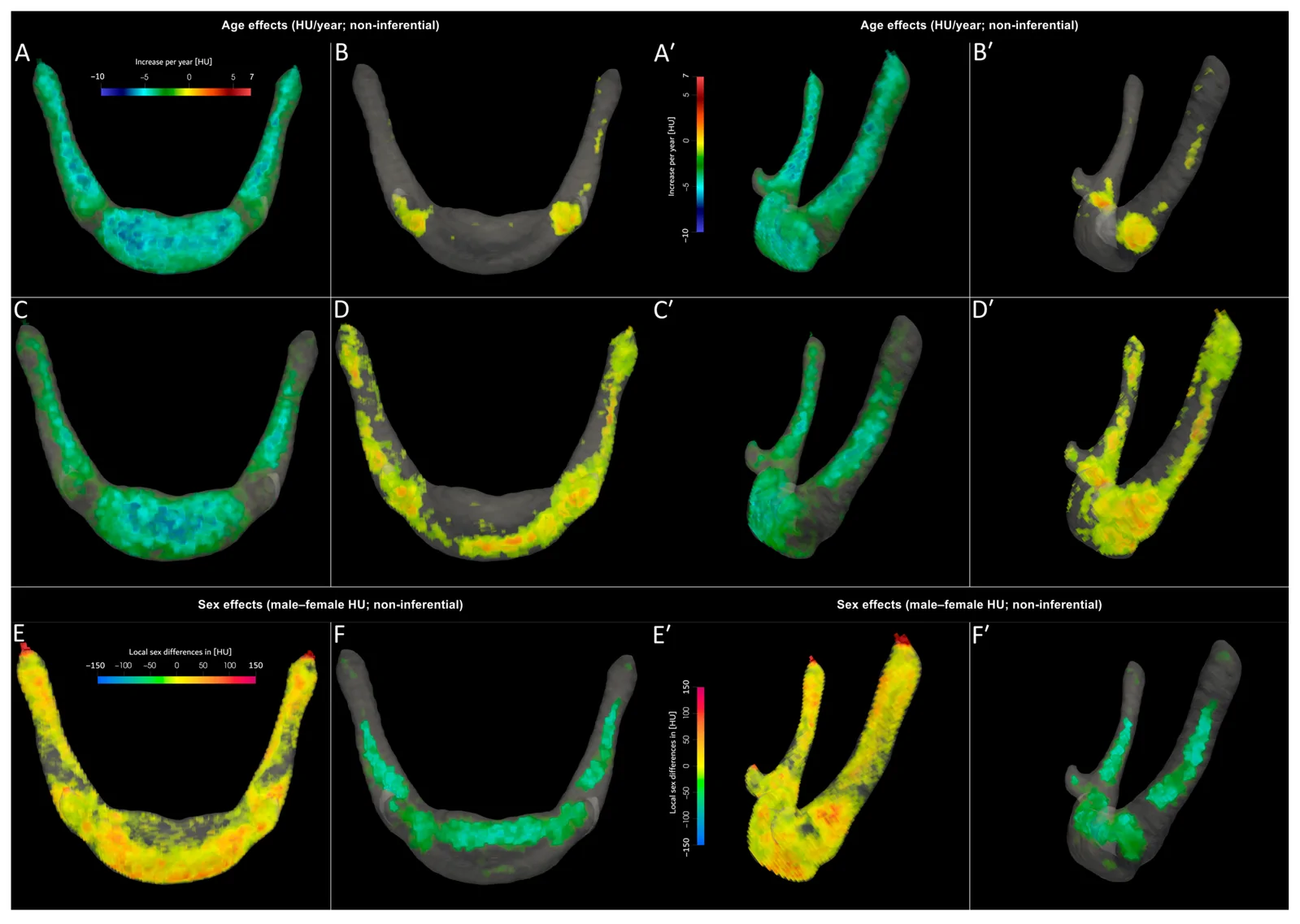
Superior and left lateral views of the hyoid template showing supervoxel-wise coefficient maps for age- and sex-related radiodensity patterns. In both views, panels (**A**,**B**) and (**A′**,**B′**) show age-related regression coefficients in females. Panel (**A**) displays negative age coefficients, corresponding to lower radiodensity with increasing age, whereas panel (**B**) displays positive age coefficients, corresponding to higher radiodensity with increasing age. Panels (**A′**,**B′**) show the corresponding negative and positive age coefficients, respectively, in the left lateral view. Panels (**C**,**D**) show the corresponding age-related coefficients in males. Panels (**C′**,**D′**) show the corresponding negative and positive age-related coefficients, respectively, in the left lateral view. Panels (**E**,**F**) show sex-related regression coefficients from the age-adjusted model. Panels (**E′**,**F′**) show the corresponding positive and negative sex-related coefficients, respectively, in the left lateral view. Positive sex coefficients in panel (**E**) indicate regions with higher radiodensity in males, whereas negative sex coefficients in panel (**F**) indicate regions with higher radiodensity in females. The hyoid body forms the anterior curved part of the template, and the greater horns extend posterolaterally. In females, negative age coefficients were broadly distributed across the hyoid body, the body–greater horn transition, and proximal-to-mid portions of the greater horns, especially along the medial surfaces facing the central opening of the hyoid. Positive age coefficients were more focal and appeared mainly near the body–greater horn transition and in restricted segments of the greater horns. In males, age-related coefficients were more spatially mixed, with negative and positive regions distributed across the body and horns without a single dominant pattern. Sex-related coefficients were regionally organized along the body–greater horn continuum. Positive sex effects were visible across large parts of the hyoid body and greater horns, whereas negative sex effects were more restricted and appeared mainly as localized bands or clusters along the body and horn shafts. Grey indicates the hyoid template surface, and colored regions show the anatomical distribution and direction of the estimated coefficients. Formal statistical inference is based on FDR-corrected supervoxel-wise results reported in the text and tables; the maps are shown to visualize the spatial pattern of the effects. The color scale for age effects applies to panels (**A**–**D**, **A′**–**D′**), whereas the color scale for sex effects applies to panels (**E**,**F**,**E′**,**F′**).

**Figure 4:**
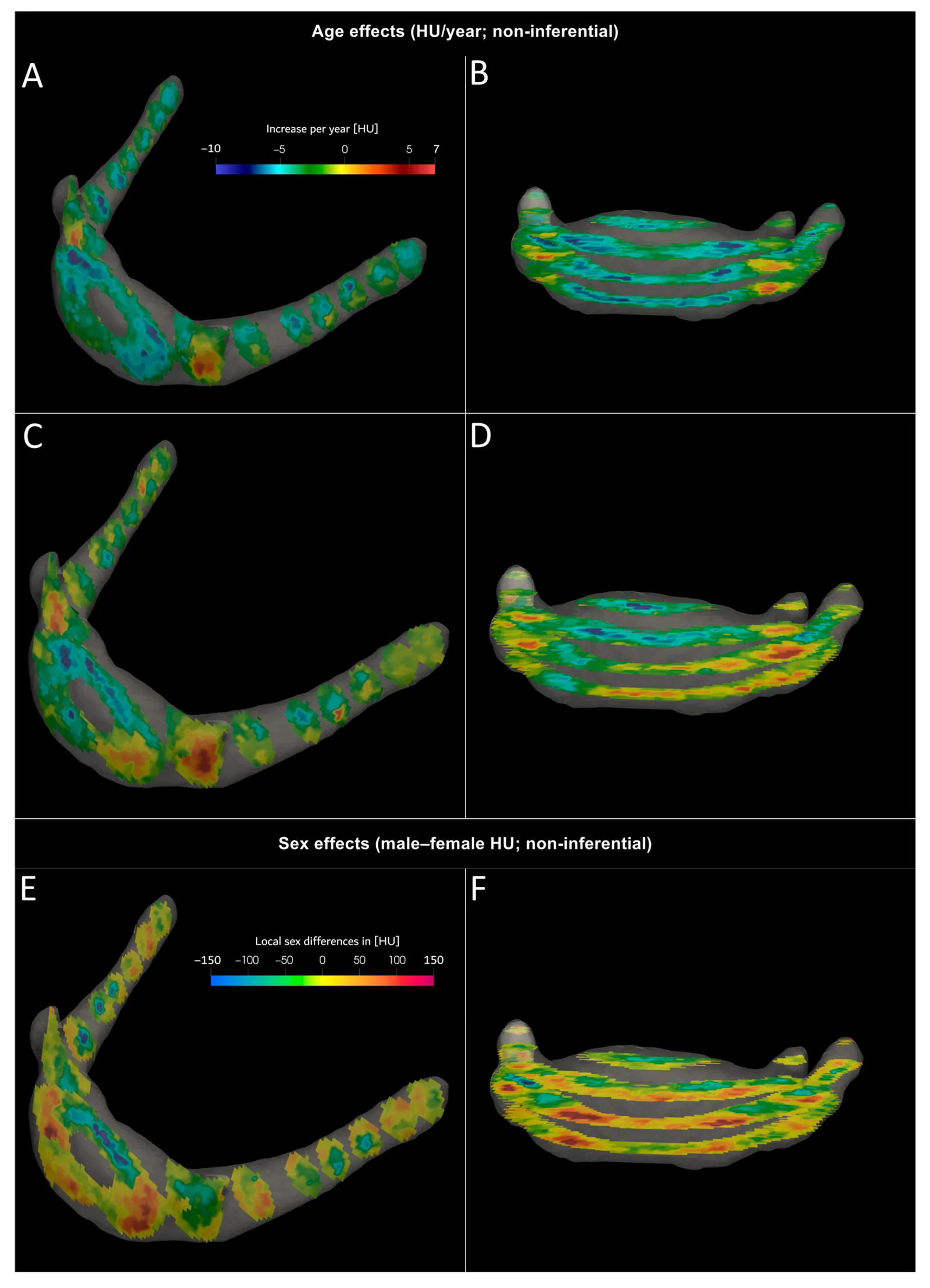
Slice-based visualization of supervoxel-wise coefficient maps within the hyoid template volume. Panels (**A**,**B**) show age-related regression coefficients in females, and panels (**C**,**D**) show age-related regression coefficients in males. In these panels, coefficient values represent the estimated change in local radiodensity in HU per year. Blue colors indicate negative coefficients, corresponding to lower radiodensity with increasing age, whereas red colors indicate positive coefficients, corresponding to higher radiodensity with increasing age. Panels (**E**,**F**) show sex-related regression coefficients from the age-adjusted model. In these panels, positive coefficients indicate regions with higher radiodensity in males, whereas negative coefficients indicate regions with higher radiodensity in females. Grey indicates the hyoid template volume, and colored regions show the spatial distribution of model coefficients within the hyoid. The slice-based view demonstrates that age- and sex-related coefficient patterns were not restricted to the external surface but extended through the hyoid body, the body–greater horn transition, and the greater horns. In females, negative age coefficients were broadly visible across the hyoid body and adjacent greater horn regions, while positive coefficients were more focal. In males, age-related coefficients showed a more mixed distribution without a clearly dominant regional pattern. Sex-related coefficients formed internal bands and clusters along the body–greater horn continuum, indicating that male–female differences were regional rather than uniform across the whole bone. Formal statistical inference is based on FDR-corrected supervoxel-wise results reported in the text and tables; the maps are shown to visualize the anatomical distribution, direction, and relative magnitude of the estimated effects. The color scale for age effects applies to panels (**A**–**D**), whereas the color scale for sex effects applies to panels (**E**,**F**).

Supervoxel-wise coefficient maps showed that age- and sex-related radiodensity effects followed recognizable anatomical regions of the hyoid rather than appearing as random or homogeneous whole-bone changes. Age-related maps in females showed the clearest spatial organization. Negative age coefficients, corresponding to lower radiodensity with increasing age, were most apparent across the hyoid body, the body–greater horn transition, and proximal-to-mid portions of the greater horns, particularly along their medial (inner) surfaces. Positive age coefficients were more limited and appeared as focal clusters within restricted parts of the body–horn continuum and greater horn segments, often extending toward the lateral (outer) aspects of the greater horns. In males, age-related coefficients were more spatially mixed, with positive and negative regions distributed across the body and greater horns without a dominant coherent pattern, although some differences between the medial and lateral surfaces of the greater horns were still evident. Sex-related coefficients were also regionally organized. Regions with higher radiodensity in males and regions with higher radiodensity in females were distributed mainly along the hyoid body, the body–greater horn transition, and longitudinal parts of the greater horns, with several of the strongest effects concentrated on either the medial (inner) or lateral (outer) surfaces rather than being uniformly distributed across the entire horn thickness. Thus, age- and sex-related variation was expressed as regional radiodensity patterning rather than as a uniform change affecting the entire hyoid equally. The sex panels show unadjusted coefficient magnitudes and directions and should not be interpreted as effects independent of native hyoid volume. Maps show unthresholded coefficient distributions for anatomical visualization; statistical significance is reported separately using FDR-corrected supervoxel-wise models. Findings at the body–greater horn junction may also reflect differences in fusion or ossification (Figure 3 and Figure 4).

## 4. Discussion

The principal finding of this study is that registered CT-derived radiodensity maps of the adult human hyoid contain spatial information that is not adequately represented by a single whole-bone HU value. In our dataset comprising 300 hyoids from adult individuals mapped into a common anatomical template, neighboring supervoxels showed more similar radiodensity profiles than distant supervoxels. This pattern may reflect both the underlying anatomy and spatial smoothing introduced by registration and interpolation. In this cohort, spatial descriptors also retained substantially more age- and sex-related information than scalar whole-bone summaries. At a global level, age was associated with lower radiodensity, but mean HU alone had poor descriptive performance for age-related variation. In contrast, PCA-based spatial descriptors reached substantially higher model performance, with R^2^ values of approximately 0.25 and Spearman correlations of 0.506 for both raw and ComBat-adjusted spatial feature sets. For sex-related variation, mean HU showed no meaningful discrimination, whereas PCA-based spatial descriptors reached ROC AUC values of approximately 0.81. Supervoxel-wise models further localized these effects anatomically. In the overall age model, 166 of 1500 supervoxels were significant after FDR correction, with a median coefficient of −1.07 HU/year. In sex-stratified models, the age-related signal was strongest in females, where 314 supervoxels were significant and the median coefficient was −1.77 HU/year, whereas no supervoxels reached FDR significance in males. Before volume adjustment, sex-related effects involved 51 significant supervoxels with a median male–female coefficient of 17.91 HU; none of these remained FDR-significant after native volume was added to the model. The strongest and most interpretable patterns involved the hyoid body, the body–greater horn transition, and the proximal-to-mid portions of the greater horns, including medial aspects of the horns and the broader body–horn continuum. Whole-bone averaging combines regions with different baseline radiodensity and potentially opposing age- or sex-related coefficients, allowing localized variation to cancel within a single summary value. Spatial descriptors retain covariation among anatomically corresponding regions and can therefore capture radiodensity redistribution that may reflect differences in mineralized tissue organization, ossification, fusion, or remodeling history, although these components cannot be separated using the present uncalibrated cross-sectional CT data.

The present findings extend previous CT and microstructural studies of the hyoid by shifting the level of analysis from global or predefined regional measurements to population-level whole-bone radiodensity mapping. Fisher et al. showed that CT-derived hyoid radiodensity decreases with age in adulthood, is lower in adult females than males, and contributes to age and sex estimation together with fusion status [26]. In contrast, our adjusted whole-bone analysis found no convincing sex effect, which may reflect differences in cohort age structure, CT acquisition, segmentation, and statistical adjustment. Werner et al. provided three-dimensional CT evidence for hyoid growth and postpubertal sexual dimorphism, including developmental changes in hyoid linear dimensions, angular measurements, and the hyoid’s relationship to the mandible and vocal tract [20]. Wang et al. used micro-CT to show that the hyoid body and greater horns differ in several internal structural parameters, including bone volume, bone density, volume fraction, trabecular connectivity, and trabecular topology, while some trabecular indices such as trabecular thickness, trabecular number, structure model index, and anisotropy did not differ significantly between these regions [28]. Kasahara et al. examined the human hyoid body histologically and found no significant regional differences in BMD across their measurement regions but observed stronger biological apatite crystallite orientation at sites associated with fibrocartilaginous entheses of infrahyoid muscles and ligaments [29]. These studies collectively show that hyoid variation cannot be reduced to external morphology or whole-bone radiodensity alone. The contribution of the present study is to bridge population-level clinical CT and regional bone-structure evidence by mapping radiodensity across anatomically corresponding regions of the whole adult hyoid. In this respect, the study applies to the hyoid the same general logic that voxel-based, atlas-based, and surface-based approaches have brought to larger skeletal elements such as the proximal femur, vertebrae, and cortical bone surfaces [15,32,33].

From a mechanic perspective, the key output of this study is not a direct measurement of strain, but an anatomical map of regions in which age and sex modulate the internal radiodensity phenotype of a force-transmitting bone. Dynamic 320-row area detector CT has shown that hyoid-related muscles do not act as a single uniform unit during swallowing: stylohyoid, posterior digastric, and mylohyoid shortenings are associated mainly with upward hyoid movement, whereas geniohyoid, thyrohyoid, and anterior digastric shortening is associated with forward movement [19]. This supports the view that the mechanical environment of the hyoid is directionally and temporally heterogeneous. The present coefficient maps are therefore biomechanically relevant at the level of localization: they identify where age- and sex-related radiodensity differences occur within the body–horn framework through which muscular and ligamentous forces are transmitted. The fact that the strongest effects involved the hyoid body, the body–greater horn transition, and adjacent horn regions is anatomically meaningful, because these regions lie within the structural continuum linking the body, horn bases, fusion zones, and attachment-related loading environments. These regions should therefore be treated as candidate targets for future biomechanical validation rather than as arbitrary statistical patches.

The age-related findings add spatial resolution to earlier observations that hyoid radiodensity decreases across adulthood [26]. In our dataset, whole-bone measures captured only a weak age association, whereas the spatial analysis localized predominantly negative age coefficients in females to the hyoid body, the body–greater horn transition, and the proximal-to-mid greater horns. The biological relevance of these modest HU/year coefficients lies primarily in their anatomical concentration rather than their absolute magnitude. They identify regions where age-associated differences occur but do not establish their underlying tissue-level cause. The coefficients at the body–greater horn junction may partly reflect whether osseous union is present; the present analysis does not separate this contribution from other sources of CT variation. Because menopausal status, hormone levels, osteoporosis diagnosis, bone-health medication, calibrated BMD, and other systemic bone-health variables were unavailable, the observed hyoid pattern cannot be attributed specifically to menopause, estrogen deficiency, or another bone condition. It is nevertheless biologically compatible with broader evidence that skeletal aging is sex dependent. Estrogen withdrawal during the menopausal transition increases bone remodeling and results in net bone loss, while longitudinal DXA studies have demonstrated accelerated BMD decline during late perimenopause and the early postmenopausal years [39,40,41,42]. However, estrogen-independent aging and skeletal-site-specific effects also contribute substantially [39]. The female–male contrast should likewise be interpreted cautiously: only 2 of 1500 formal age × sex interactions survived FDR correction, and more than one quarter of female samples downsampled to *n* = 120 produced no FDR-significant findings despite retaining similar coefficient directions. Thus, the difference between 314 female and 0 male discoveries does not establish a widespread sex-specific difference in age-related change and was influenced by subgroup size and multiple-testing precision.

Because age was modeled as a continuous adult variable, the present analysis was also not designed to identify threshold ages or nonlinear transitions. Future studies with larger age-stratified cohorts, explicit modeling of fusion status, and clinical bone-health metadata will be needed to determine whether specific adult life stages are associated with shifts in the spatial hyoid radiodensity phenotype.

In our dataset, sex-related variation was captured by both native hyoid size and spatial radiodensity. Native volume alone classified sex with an AUC of 0.909, whereas adjustment of the spatial features for volume reduced their AUC from 0.810 to 0.671; none of the 51 original regional sex associations survived volume adjustment. Much of the sex-related spatial signal was therefore size-linked, potentially reflecting both true morphological dimorphism and size-dependent partial-volume effects. Because the adjusted classifier remained above chance, volume did not explain all spatial information, but the present data do not establish a localized sex-related radiodensity pattern independent of size. This interpretation is consistent with previous evidence that hyoid morphology, growth, fusion, and CT-derived radiodensity characteristics differ between males and females [20,26], while showing that these properties are closely interrelated in clinical CT. For a bone with marked variation in size, curvature, horn morphology, fusion status, and muscle–ligament attachment environments, spatial averaging may therefore obscure the regional variation most relevant for structure–function interpretation. With external validation, spatial radiodensity mapping may complement established morphometric and imaging-based approaches to biological profile estimation by adding information about internal regional organization that is not captured by conventional dimensions or whole-bone HU summaries.

Methodologically, this study shows that template-based radiodensity mapping is feasible even in a small and morphologically variable skeletal element. Mapping all hyoid bones into a common template space allowed local radiodensity to be compared region by region, while the supervoxel representation reduced voxel-level noise and produced a manageable spatial feature set. Because the template and supervoxel partitioning were derived once from the full cohort, the resulting representation was not fully independent of the held-out cases and may therefore favor within-cohort performance, even though no age, sex, or predictive outcome information was used. The low degree of missingness after aggregation and the distance-dependent correlation structure indicate that the registered representation was spatially coherent, although this coherence may partly reflect registration, interpolation, and anatomical smoothness. Accordingly, adjacent FDR-significant supervoxels are best interpreted as parts of continuous regional patterns rather than as independent biological discoveries. The reported counts therefore describe regional tests passing the BH-FDR threshold, whereas anatomical interpretation is based on their broader spatial distribution and the candidate regions they collectively identify. The similarity between raw and ComBat-adjusted PCA model performance further suggests that the strongest spatial signal was not merely an acquisition artifact, although technical variation remained relevant and required explicit handling. This is central for studies using routine clinical CT. The present data were not calibrated QCT, but the acquisition context was comparatively controlled: scans came from a single CT system, used a consistent 120 kV protocol and H60s reconstruction kernel, and were analyzed at a common voxel size of 0.508 × 0.508 × 0.600 mm. Technical covariates and harmonization were therefore used not to claim absolute BMD measurement, but to support cautious interpretation of HU as relative radiodensity within a standardized analytical framework.

Several limitations define the boundary of inference. First, the study used routine uncalibrated clinical CT. HU values should therefore be interpreted as relative radiodensity, not as calibrated BMD or direct tissue mineral density. HU values are affected by scanner properties, acquisition settings, reconstruction kernel, spatial resolution, beam hardening, segmentation, registration, interpolation, and partial-volume effects. Segmentation agreement was available only for the whole hyoid because the reference masks did not contain validated body and greater-horn labels. These issues are particularly relevant because hyoid size affects the proportion of boundary voxels; consequently, the unadjusted sex maps may reflect both true morphological dimorphism and size-dependent partial-volume effects, which cannot be separated in the present data. At the same time, the standardized acquisition setting, common preprocessing, technical covariate control, ComBat-based harmonization of spatial descriptors, and FDR-corrected regional analyses reduce the likelihood that the age-related findings are explained entirely by acquisition-related variation. Second, the study was cross-sectional, so age-related differences describe variation across individuals rather than longitudinal change within the same individuals. Third, the study did not measure muscle force, swallowing kinematics, speech function, airway mechanics, histology, biological apatite orientation, cortical porosity, trabecular microarchitecture, or finite element strain. The present data therefore do not prove that specific radiodensity regions are caused by local muscle loading or functional adaptation. This limitation does not remove the biomechanical relevance of the study; rather, it defines the level at which biomechanical inference can be made. The study identifies candidate regions where age and sex influence the spatial radiodensity phenotype of a force-transmitting bone. The most direct next step is to test these regions against micro-CT, histological enthesis mapping, biological apatite orientation, calibrated or phantomless QCT, and finite element models based on realistic supra- and infrahyoid loading conditions. Integration with dynamic swallowing or speech imaging would further clarify whether local hyoid radiodensity patterns relate to individual variation in hyolaryngeal movement and force transmission.

## 5. Conclusions

This study established a template-based framework for spatial radiodensity mapping of the adult human hyoid from routine clinical CT. By normalizing 300 hyoids into a common anatomical space, we enabled region-to-region comparison of CT-derived radiodensity across a small and morphologically variable bone.

The registered radiodensity maps showed spatial coherence, with neighboring supervoxels having more similar profiles than distant ones; this pattern may partly reflect registration, interpolation, and anatomical smoothness. Spatial descriptors captured age-related variation more effectively than conventional whole-bone HU summaries. They also captured sex-related variation, but this was substantially attenuated after accounting for native hyoid volume. These findings remain to be confirmed in a cohort not used to construct the template.

Regional analyses identified the hyoid body, the body–greater horn transition, and the proximal-to-mid greater horns as candidate regions for future investigation of age-associated radiodensity patterns. The registered hyoid radiodensity maps were spatially coherent, but the observed sex-related pattern is strongly coupled to bone size and requires confirmation after explicit control of morphology and partial-volume effects. Although routine uncalibrated CT cannot provide calibrated BMD or direct biomechanical measurements, template-based hyoid radiodensity mapping provides a practical spatial baseline for future validation against microstructure, enthesis morphology, fusion status, and biomechanical models of supra- and infrahyoid loading.

## Figures and Tables

**Table 1:** Comparative performance of scalar and spatial descriptors for age-related variation. Values are pooled out-of-fold estimates ± SD across the five folds.

Feature Set	MAE (Years)	RMSE (Years)	R^2^	Spearman Rho
Mean HU	10.76 ± 1.22	13.86 ± 1.27	−0.011 ± 0.064	0.025 ± 0.098
Raw whole-bone summary	10.70 ± 1.19	13.69 ± 1.32	0.013 ± 0.117	0.159 ± 0.176
ComBat-adjusted summary	10.44 ± 0.79	13.14 ± 0.95	0.090 ± 0.097	0.283 ± 0.199
Raw PCA from supervoxels	8.91 ± 1.03	11.89 ± 1.27	0.255 ± 0.078	0.506 ± 0.054
ComBat-adjusted PCA supervoxels	8.96 ± 1.04	11.92 ± 1.22	0.252 ± 0.060	0.506 ± 0.053

**Table 2:** Comparative performance of scalar and spatial descriptors for sex-related variation. Sex was coded as female = 0 and male = 1, with male as the positive class; sensitivity therefore refers to males and specificity to females. Values are pooled out-of-fold estimates ± SD across the five folds.

Feature Set	ROC AUC	Balanced Accuracy	Sensitivity (M)	Specificity (F)
Mean HU	0.471 ± 0.075	0.481 ± 0.041	0.450 ± 0.090	0.511 ± 0.064
Raw whole-bone summary	0.607 ± 0.057	0.579 ± 0.053	0.508 ± 0.068	0.650 ± 0.089
ComBat-adjusted summary	0.607 ± 0.051	0.578 ± 0.053	0.500 ± 0.072	0.656 ± 0.078
Raw PCA from supervoxels	0.810 ± 0.067	0.725 ± 0.047	0.700 ± 0.062	0.750 ± 0.052
ComBat-adjusted PCA supervoxels	0.808 ± 0.067	0.729 ± 0.053	0.708 ± 0.066	0.750 ± 0.052

**Table 3:** Summary of supervoxel-wise regional effects in the primary models without native-volume adjustment.

Map	Median Effect	Significant Supervoxels, *n*	Significant Supervoxels, %
Age effect, all subjects	−1.07	166	11.1
Age effect, females	−1.77	314	20.9
Age effect, males	−0.46	0	0.0
Sex effect (M-F)	17.91	51	3.4

## Data Availability

The individual clinical CT datasets used in this study are not publicly available because they contain sensitive personal health information and are subject to data protection and institutional privacy restrictions. Informed consent did not include permission for public sharing of individual-level CT data, but allowed the sharing of derived, non-identifying analytical outputs. Derived template-space geometries, spatial statistical outputs, and the Python script describing the complete analytical workflow are available as Supplementary Material via Figshare https://doi.org/10.6084/m9.figshare.32820437 (accessed on 24 August 2026).

## Supplementary Materials

The following supporting information can be downloaded at: https://doi.org/10.6084/m9.figshare.32820437 (accessed on 24 August 2026): template-space geometry with the spatial statistical maps presented in Section 3.6, accompanying README files, and the Python script documenting the complete analytical pipeline.

## Abbreviations

The following abbreviations are used in this manuscript:

ANTsPy	Advanced Normalization Tools in Python
APC	Article processing charge
AUC	Area under the curve
BH-FDR	Benjamini–Hochberg false discovery rate
BMD	Bone mineral density
ComBat	Empirical Bayes batch harmonization method
CT	Computed tomography
DSC	Dice similarity coefficient
DXA	Dual-energy X-ray absorptiometry
FDR	False discovery rate
HD95	95th-percentile Hausdorff distance
HU	Hounsfield unit
IQR	Interquartile range
KNN	k-nearest neighbors
LLM	Large language model
MAE	Mean absolute error
PCA	Principal component analysis
QCT	Quantitative computed tomography
RMSE	Root-mean-square error
ROC	Receiver operating characteristic
SD	Standard deviation
